# Enhancing heart disease prediction using a self-attention-based transformer model

**DOI:** 10.1038/s41598-024-51184-7

**Published:** 2024-01-04

**Authors:** Atta Ur Rahman, Yousef Alsenani, Adeel Zafar, Kalim Ullah, Khaled Rabie, Thokozani Shongwe

**Affiliations:** 1 Riphah Institute of System Engineering, Riphah International University Islamabad, Islamabad, 46000 Pakistan; 2 Department of Information Systems, FCIT, King Abdulaziz University, 21443 Jeddah, Saudi Arabia; 3 Research and Development Department, Lun Startup Studio, 11543 Riyadh, Saudi Arabia; 4 Department of Zoology, Kohat University of Science and Technology, Kohat, 26000 Pakistan; 5 Department of Engineering, Manchester Metropolitan University, Manchester, M15 6BH UK; 6 Department of Electrical and Electronic Engineering Science, University of Johannesburg, Johannesburg, 2006 South Africa

**Keywords:** Computational biology and bioinformatics, Mathematics and computing

## Abstract

Cardiovascular diseases (CVDs) continue to be the leading cause of more than 17 million mortalities worldwide. The early detection of heart failure with high accuracy is crucial for clinical trials and therapy. Patients will be categorized into various types of heart disease based on characteristics like blood pressure, cholesterol levels, heart rate, and other characteristics. With the use of an automatic system, we can provide early diagnoses for those who are prone to heart failure by analyzing their characteristics. In this work, we deploy a novel self-attention-based transformer model, that combines self-attention mechanisms and transformer networks to predict CVD risk. The self-attention layers capture contextual information and generate representations that effectively model complex patterns in the data. Self-attention mechanisms provide interpretability by giving each component of the input sequence a certain amount of attention weight. This includes adjusting the input and output layers, incorporating more layers, and modifying the attention processes to collect relevant information. This also makes it possible for physicians to comprehend which features of the data contributed to the model's predictions. The proposed model is tested on the Cleveland dataset, a benchmark dataset of the University of California Irvine (UCI) machine learning (ML) repository. Comparing the proposed model to several baseline approaches, we achieved the highest accuracy of 96.51%. Furthermore, the outcomes of our experiments demonstrate that the prediction rate of our model is higher than that of other cutting-edge approaches used for heart disease prediction.

## Introduction

Heart disease refers to any condition that impairs the heart’s capacity to function normally. In recent years, CVD has become the leading cause of death in the world. Congestive heart failure (CHF) prevalence is expected to rise by 46% by 2030 compared to 2012 rates^1^. The incidence and mortality rates of CVD can be significantly lowered by diagnosing the problem, according to research, in both patients who are already aware of their condition and those who are not^2^. Early detection and diagnosis can result in prompt interventions and suitable therapies, which can enhance patient outcomes and lower the chance of problems. The successful diagnosis of cardiac abnormalities and valve heart disorders (VHDs) in recent years has been made possible by the use of phonocardiogram (PCG) data in combination with ML techniques. These algorithms use a variety of feature extraction methods and classifiers to precisely identify and diagnose cardiac problems^3^. Traditional ML methods have numerous drawbacks despite their potential. The methods frequently lack precision and robustness, which can result in false positive or false negative results^4^. The iterative nature of feature selection and classifier optimization procedures can frequently take a lot of time, which can impede the prompt diagnosis and effective treatment of cardiac disease^5^. Deep learning (DL) algorithms, supported by big-data techniques, have become an effective tool for identifying and recognizing cardiac disease in order to get around these restrictions. In many different fields, including image classification, computer vision, object localization, electroencephalogram (EEG) signal classification for brain-computer interfaces, and physics-informed neural networks, among others^6^, DL algorithms have achieved remarkable success. They can automatically extract non-linear and hierarchical features from large datasets^7^. We may be able to increase the reliability and accuracy of cardiac disease detection and diagnosis, as well as promote quick interventions and treatments for better patient outcomes, by utilizing recent developments in DL algorithms and big data methodologies. Despite their potential, DL models can be computationally expensive and take longer to train^8^, which may restrict their usefulness in the detection and diagnosis of heart disease. The Vision Transformer (ViT), a recent advancement in DL, has demonstrated encouraging results in resolving these difficulties^9^. By utilizing the self-attention strategy to get over image-specific biases and constraints, ViT has shown greater accuracy and computational efficiency when compared to state-of-the-art Convolutional Neural Network (CNN) models^10^. DL-based algorithms have demonstrated good efficiency in categorizing heart sounds for VHD, but they frequently suffer from insufficient deep spatial feature extraction, leading to decreased accuracy^11^. Additionally, the high computational costs and lengthy training times associated with DL models can make it more difficult to improve heart sound classification ability^12^.

Patients with heart failure and society as a whole would benefit from accurate, organized diagnostic services^13^. In order to do this, this study creates a novel method for performing heart disease prediction by utilizing an improved self-attention-based transformer network. Preprocessing the dataset includes dealing with missing values, encoding category variables, and normalizing numerical characteristics. Extract significant features from the dataset, such as age, gender, blood pressure, cholesterol levels, etc. The model architecture is fine-tuned by utilizing several attention layers, feed-forward neural networks, positional encodings, etc. We noticed improved diagnosis by doing experiments on a benchmark dataset. The study conducted in^14^ found that extracting relevant information is the most important step in improving the precision of heart disease detection. For example, a clinician makes a decision on a patient with heart disease based on the classification using the specified characteristics. Previous research focused on enhancing and creating classification techniques rather than choosing the optimal attributes and their relationship to increase accuracy^15^. Using the self-attention mechanism, the proposed model can effectively capture the relationships and dependencies between distinct features in the data. This allows the transformer to focus on critical information while downplaying less significant aspects, improving the model’s capacity to extract important patterns and information.

The remainder of the paper is organized as follows: “Related work” section provides an overview of the related work. In “Limitation and motivation” section, we delve into the background and motivation. The detailed problem description is presented in “Proposed framework” section. The experiments conducted in this work are discussed in “Experiments” section. The results and discussion are explained in “Results and discussion” section. Finally, “Conclusions” section concludes this work and gives future directions.

## Related work

Heart disease is one of the primary reasons for mortality worldwide. With the use of Artificial Intelligence (AI) approaches, it is possible to monitor certain characteristics such as blood pressure, body weight, cholesterol, sugar level, and heart rate to determine cardiac disease in its initial stages. ML and DL techniques are revolutionizing the current healthcare system however it is challenging to predict cardiac disease accurately and reliably^16^. Various classification methods have been utilized for heart disease prediction. The ensemble learning algorithm, in particular Random Forest (RF), has shown some good results in predicting heart disease^17^. The study conducted in^18^ used support vector machines (SVM) for classification after using feature selection methods such as the Fisher score and Matthew’s correlation. A DL system called DeepLabeler was created in the study conducted in^19^ to automatically classify ICD-9 codes. Their developed system uses the document-to-vector (D2V) method and a CNN to capture and encode both local and global data. The model’s two key characteristics are multi-label classification and feature extraction. The Reverse Time Attention model (RETAIN), which incorporates an attention mechanism and is based on a combination of Recurrent Neural Networks (RNNs), was used in the study conducted in^20^. This allows the model to focus on the most significant attributes or time periods in the input sequence. The understanding of RETAIN is improved by giving each characteristic or time step in the sequence a weighted relevance score. In this way, the clinicians and experts can then understand what factors or time sequences are most crucial for the model’s predictions. Current cutting-edge DL models lack excellent feature extraction capabilities in complicated and noisy situations, restricting the development of precise and consistent object differentiation^21^. The previous research may be broadly divided into two categories: DL approaches and classic shallow approaches^22^.

For the precise diagnosis of valve heart diseases (VHDs), a robust and high-performing DL model has been provided in^23^. The study published in^24^ developed a model for forecasting the possibility of CVD in their sample utilizing data from a Japanese urban cohort study. The system for the diagnosis of coronary disease and stroke was constructed using multivariable Cox proportional hazard methods. They were able to examine a variety of factors and produce a reliable model for assessing the risk of CVD events by using their suggested technique. A unique ML method for heart disease prediction was created in the research reported in^25^. They applied RF and Decision Tree (DT) approaches using the Cleveland heart disease dataset. Their experimental findings showed an accuracy of 88.7% for identifying heart disease. Numerous ML techniques were applied to evaluate massive and complicated medical data, assisting healthcare professionals in the early diagnosis of heart disease^15^. The study employed a number of classification models, including DT, Naive Bayes (NB), K-nearest Neighbour (KNN), and RF algorithm, to compute a variety of heart disease-related problems. Their study’s main goal was to estimate the probability of people having a chance of heart attacks in the future.

With the help of sequential electronic health record (EHR) data, the study conducted in^13^ attempted to diagnose cardiac failure. They made use of real-world datasets that contained data from hospital departments, health records, and patient diagnostic information pertaining to cardiac diseases. The main aim of their study was to precisely detect and classify individuals at risk of heart failure by the analysis of comprehensive EHR data. The efficiency of merging tree-based ensemble methods with the Synthetic Minority Over-sampling Technique (SMOTE) was conducted^26^. This method was used to deal with the problem of data imbalance in heart failure patient survival prediction. The study aims to maximize the accuracy of forecasting the survival outcomes for patients with heart failure by using ensemble methods and applying SMOTE to rebalance the data. The study conducted in^27^ deployed a hybrid model incorporating clustering and classification in the field of type 2 diabetes prediction. K-means clustering was the first phase in this model’s two-step process, which was followed by the C4.5 classification technique using a k-fold cross-validation approach. The proposed hybrid approach produced encouraging results, with a classification rate of 88.38%. The use of this model has enormous potential for doctors since it can help them make well-informed clinical decisions about the management of diabetes.

The most current research demonstrates the various methods used to increase heart disease prediction accuracy. Researchers have made tremendous progress in improving the precision and effectiveness of prediction models through the use of ensemble learning^28^, feature extraction^29^, DL models^30^, and other techniques. In order to overcome the limitations of earlier work, a unique method of heart disease prediction is presented, utilizing a self-attention-based transformer model. This cutting-edge model was created expressly to solve the difficulties in investigating and forecasting cardiac disease. The model successfully captures complex patterns and relationships within the medical data by utilizing self-attention processes, allowing for more precise predictions.

## Limitation and motivation

Statistics of heart disease often include temporal characteristics, such as the history of the patient as well as variations over time. Effectively processing sequential data using ML approaches is challenging. Previous studies didn’t provide sufficient support for better patient outcomes. In this section, we outline the limitations of previous heart disease prediction methods, clarify our work motivations for developing an improved model, and highlight the key contributions and novelties of our study.

### Previous works limitations

The primary input sources for heart disease diagnosis are patient health characteristics containing data with categories and unstructured text. The main shortcomings of the current heart disease prediction methods are the modeling of input dataset attributes, computation of attribute risk factors, and obtaining high prediction accuracy^31^. The significant drawback of NB in the context of heart disease prediction is that it treats each feature of the dataset individually when calculating probabilities. Therefore, conventional classifiers lead to an incorrect decision support system^32^. According to earlier research, traditional medical decision support systems often focused solely on increasing classification accuracy. They failed to consider the varying costs of misclassification across other categories. However, the minority class frequently has a higher priority in the field of healthcare decision making. The efficiency of RNN-based models tends to deteriorate rapidly as data sequence length increases. They perform poorly because of their sequential character, which prevents them from correctly capturing long-term relationships within the data sequences^33^.

Traditional RNNs are prone to vanishing and expanding gradient problems. The Standard Long Short-Term Memory (LSTM) networks have the drawback of being unable to handle irregular periods of time. However, timing inconsistency is typical in many healthcare applications^34^. By incorporating an attention-based mechanism that makes it possible to effectively capture dependencies, enhance interpretability, and enable computation parallelization, the proposed model seeks to reduce the limitations of the previous work.

### Motivation

Disease prediction systems are best practices for eliminating human errors in disease diagnosis and aiding in disease prevention through early identification^31^. Diagnosis of cardiac disease based on patient health record characteristics is a multidimensional decision-making technique. Prediction of heart disease is crucial for healthcare since it may improve patient outcomes significantly when it is detected early and accurately. However, there are certain issues with adaptability, interpretability, and training speed in the existing prediction model. This work created a cutting-edge and reliable attention-based model for heart disease prediction in order to overcome the difficulties of the previous work. The proposed model has the potential to quickly and readily adapt to different outcome risk prediction and evaluation challenges, which makes it a useful tool in the field of healthcare prediction^35^.

Furthermore, the proposed model has a straightforward and parallelizable network structure, which leads to noticeably quicker training times than existing heart disease prediction techniques. This enhancement makes them more efficient by addressing the difficulties associated with model training and implementation in actual healthcare settings.

### Key contributions and novelty

This study presents a novel prediction model that makes use of the self-attention process. The model is created with interpretability and parallelizability in mind, enabling effective computing while maintaining a respectable level of prediction accuracy. A key element of our model is self-attention, which is notably influenced by the work done in^6^. Through the establishment of clear linkages between events, the self-attention mechanism enables us to identify dependencies within the features. It’s noteworthy that the self-attention mechanism constantly captures the weight of feature values, even when they are not independent. The final representation vector is created by adding a position-level attention layer. We employ a padding-mask method in both the self-attention and position-level attention processes to account for the variation in sequence lengths. Masking away the padding elements during the attention computation, this makes guarantees that the model can handle sequences of various lengths well. The major technical contributions of our study are summarized as follows:

- Developed an innovative and resilient attention-based model specifically tailored for predicting heart disease. In addition to its exceptional accuracy in prediction, this model also displays its adaptability to a variety of other risk prediction and evaluation tasks. Due to its adaptability, it can be used well across a variety of domains, making it an important tool for many different outcome prediction issues. Its versatility makes it suitable for various healthcare scenarios and expands its potential to tackle a broad range of predictive tasks beyond heart disease prediction.
- Investigate the key factors that lead to the risk of developing heart disease and identify any previously unknown risk factors that may be relevant.
- Design a Transformer model-based strategy that is more precise and successful than current conventional ML models in forecasting the likelihood of heart disease.
- The Transformer model’s efficiency in detecting the likelihood of heart disease across multiple demographic categories, such as age, gender, and race/ethnicity, is examined, and the possibility for personalized risk assessment is also investigated.
- The ability of the Transformer model to identify potential cardiac disease was examined in relation to the impacts of various data preprocessing techniques. Several pre-processing methods were applied to the input data, and their effects on the model’s functionality and accuracy were carefully examined.

## Proposed framework

The goal of this research is to develop a self-attention-based transformer model for assessing CVD risk utilizing the Cleveland dataset. This dataset contains a variety of medical and non-medical components that can be used to identify whether a patient has cardiac disease. The dataset comprises both continuous and categorical variables, among other features. It becomes challenging to identify the most important factors and comprehend their relevance in heart disease prognosis. Furthermore, it might be challenging to draw meaningful findings since some of the features are challenging to evaluate clinically.

### Dataset and preprocessing

In this work, we predict cardiac disease using the UC Irvine Cleveland dataset^36^. The collection consists of 303 cases, each of which depicts a patient who may have heart disease. The dataset is generated from actual patients with suspected cardiac disease, making it applicable to real-world circumstances. The information comprises a number of characteristics that are often utilized in clinical practice, including age, cholesterol levels, and electrocardiogram (ECG) readings. Each instance has 14 features that represent distinct characteristics of the patient and diagnostic measures. In the dataset, each row corresponds to a patient, and the columns represent several attributes related to the diagnosis of heart disease. The column consists of [’Age’, ’Sex’, ’Cp’, ’Trestbps’, ’Chol’, ’Fbs’, ’Restecg’, ’thalach’, ’Exang’, ’Oldpeak’, ’Slope’, ’Ca’, ’Thal’, and ’Target’]. Based on the provided attributes, the dataset is utilized to create prediction models that estimate the chance of heart disease. The dataset has been preprocessed to handle missing values, normalize numerical features, and encode categorical variables. To find any missing values, examine each characteristic. Replace the missing data with approximated values, such as mean and median. The Z-score, which estimates a data point’s deviation from the mean value, reflects the variation of an attribute’s value within a dataset. With the help of this method, we were able to successfully recognize and manage extreme values in the data.

1 $${Z}_{{\text{score}} \, }=\frac{x-\mu }{\sigma }$$

### Attention-based model architecture

**Figure 1:**
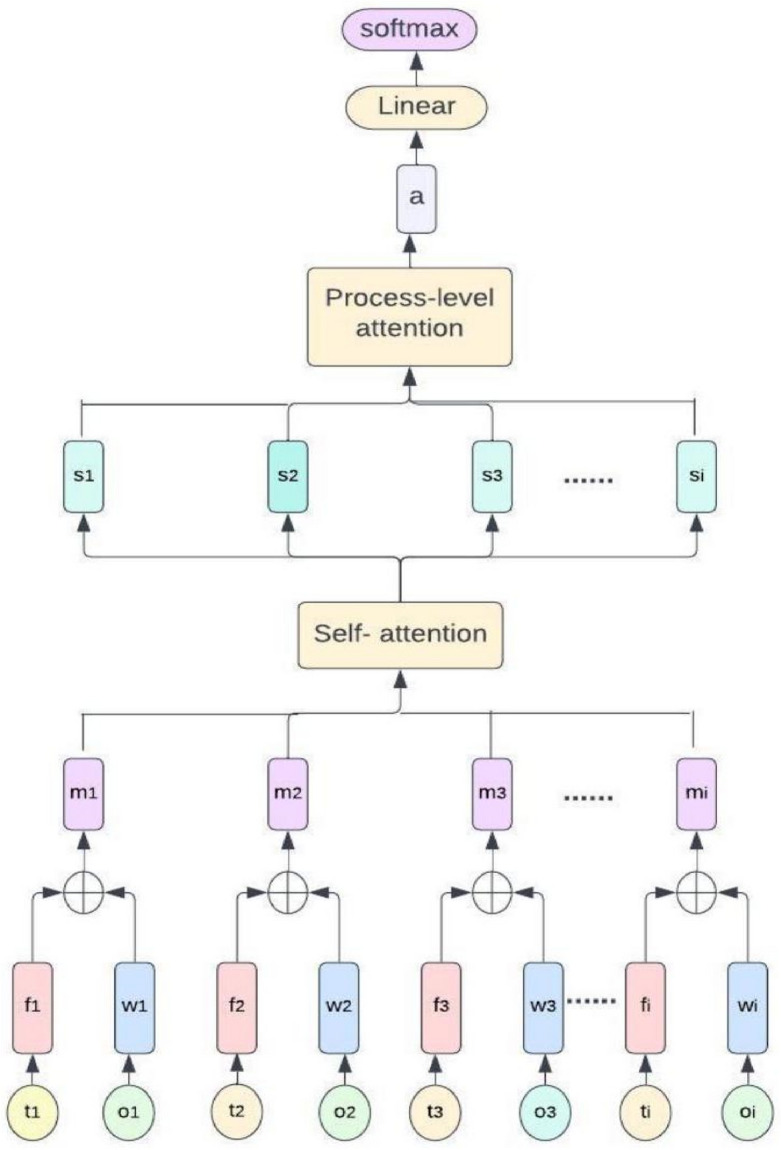
Overview of the proposed model.

The patient characteristics and diagnostic measures are represented by a series of input features that are used to encode each instance in the dataset. In this work, we have $X=\left[{x}_{1},{x}_{2},\dots ,{x}_{n}\right]$, represent the input sequence of features, where n denotes the length of the sequence. The self-attention mechanism recognizes the relationships between various aspects in the sequence and gives each feature a weight based on how important it is in relation to other features. To capture various sorts of interactions and improve model performance, several parallel self-attention layers are used. To identify non-linear interactions and provide final predictions, the attention outputs are fed into a feed-forward neural network. This attention-based model architecture with self-attention and multi-head attention mechanisms efficiently captures connections and dependencies within the input sequence, allowing the model to focus on key aspects for heart disease prediction. Figure 1 represents the visual description of the proposed model.

### Input embedding and position encoding

In the self-attention-based transformer model, input embedding and position encoding are two crucial processes that come before the self-attention mechanism. The input sequence is represented using these stages in a way that is appropriate for the successive self-attention layers. The categorical variables and numerical characteristics of each instance are translated to continuous vector representations through input embedding. In this study, we use an embedding layer to convert discrete values for each category variable into continuous vectors. Each instance's scaled numerical characteristics and category embeddings are combined into a single vector. We have $e\left({x}_{i}\right)$ denotes the embedding of instance $\left({x}_{i}\right)$ and $f\left({x}_{i}\right)$ represents the scaled numerical features of $\left({x}_{i}\right)$. The concatenated input embedding for each instance $\left({x}_{i}\right)$ is computed as: ${x}_{i}^{\mathrm{^{\prime}}}=\left[e\left({x}_{i}\right),f\left({x}_{i}\right)\right]$. The model comprehends the order or sequence of the instances by using position encoding, which adds positional information to the input sequence. The self-attention-based transformer model efficiently processes the input sequence, collecting both feature representations and positional information by executing input embedding and position encoding stages.

### Transformer encoder

The heart disease dataset is represented as a series of embedded characteristics and positional encodings. To identify relationships and extract meaningful representations from the input sequence, apply a stack of Transformer encoder layers. The encoder layer includes a self-attention mechanism and a feed-forward neural network. The output of the Transformer encoder layer is computed as, $E(i)=\left[{e}_{1}(i),{e}_{2}(i),\dots ,{e}_{n}(i)\right]$, where each $e(i)$ represents the output representation for the corresponding position in the sequence.

### Self-attention

The ability of the Transformer model to find links between features that go beyond sequence adjacency is another intriguing feature of this system. The self-attention technique is utilized to extract the relationships between various points in the sequence inside each Transformer Encoder layer. The similarity between the query and key vectors is used to determine the attention weights (AW) for each point. These AWs illustrate the relative importance of each position. AW can be calculated as follows:

2 $${\text{A}}W={\text{soft}}-{\text{max}}\left(\frac{{Q}_{u}^{R}{K}_{e}}{\sqrt{dk}}\right)$$

${Q}_{u}$ and ${K}_{e}$ are the query and key correspond to input embedding $\left({e}_{1},{e}_{2},\dots ,{e}_{i}\right)$. Following that, utilizing the attention weights matrix ${\text{AW}}$, we construct a weighted sum of the value vectors as the latest value vectors:

3 $${\text{Attention}}\left({\text{AW}},{{\text{V}}}_{a}\right)={\text{AW}}\cdot {\text{V}}$$

where ${V}_{a}$ represents the input embeddings. Additionally, we address the issue of the sequences' variable lengths by employing the same padding mask technique as the Transformer^6^. By extending the provided information in the $y$ direction back to the input $x$, the straightforward nature of the model allows us to easily determine the impact of each feature. Through the use of an embedding layer, we are able to grasp the essence of each feature in the given input sequence $x$.

4 $${E}_{vi}={V}_{vi}\cdot x$$

The learning parameters ${V}_{vi}$ and the learned visited embedding ${E}_{vi}$ are involved in the process. we introduce an additional embedding layer that specifically encodes the order information. This layer serves the purpose of preserving and incorporating sequential information into the model.

### Feed-forward network

To further enhance the representations, follow the self-attention strategy by applying a feed-forward neural network to each point separately. A non-linear activation function separates the two linear layers that make up the feed-forward network. Connect the input characteristics to the output of the self-attention mechanism and the output of the feed-forward network to create residual connections. The features after each sublayer are normalized using the layer normalization method.

### Output layer

To detect the existence of heart disease, use the final output from the Transformer Decoder layers and feed it through a fully connected layer. To determine the final output probabilities, use the softmax function.

5 $$y={\text{softmax}}\left({V}_{a}+e\right)$$

### Training the model

The Adam optimizer is used as an optimization technique to train the model for determining the likelihood of heart disease. To find whether a subject has heart disease or not, the binary cross-entropy loss function is employed to distinguish between the predicted probabilities and the actual data labels. The training method seeks to identify the ideal values for the weight vector W and the bias term b that minimize the loss function. The prediction accuracy of the model is enhanced by the Adam optimizer, which iteratively modifies the weights and biases during training.

### Disease prediction

**Figure 2:**
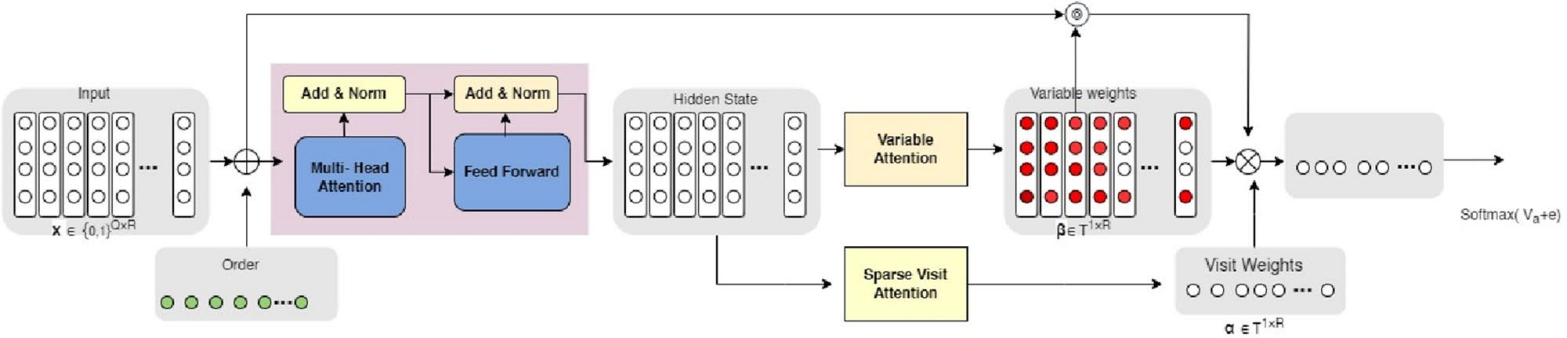
Architecture of proposed model used for heart disease prediction.

Using the test dataset, evaluate the trained model using relevant evaluation measures including accuracy, precision, recall, and F1-score. Using the trained model, forecast the likelihood that a new patient will be diagnosed with heart disease based on the feature values of the patient. Analyze the feature importance or coefficients learned by the model to identify the relative importance of different factors in determining heart disease. Figure 2 represents the proposed model for heart disease prediction.

## Experiments

Heart disease is a prominent cause of death globally, and effective prediction of heart disease can considerably improve patient outcomes^15^. In this work, we suggest using a Self-Attention-based Transformer Model to improve heart disease prediction. We make use of the Cleveland dataset^36^, a frequently used benchmark dataset in the field of cardiovascular research, to assess the effectiveness of our proposed approach. Load the Cleveland dataset into a Data Frame by using the Panda’s package. The dataset has 303 samples, each of which has 76 attributes. These characteristics include data on the patient’s demographics, health metrics, and diagnostic results. The panda’s function fillna is used to handle missing values, while StandardScaler from scikit-learn is used to normalize the dataset. The preprocessed dataset is partitioned into 80% training, 10% testing, and 10% validation sets. Using the proposed framework, we represent each sample in the dataset as a series of feature vectors, with each feature vector representing a different characteristic. To preserve the sequential information, we use positional encoding. Utilize the self-attention technique to enable the model to focus on various input sequence components while producing context-aware representations for each attribute. Create the model architecture by specifying the essential layers, such as self-attention, feed-forward, and classification layers. Use the PyTorch 2.0. framework to implement the Self-Attention-based Transformer model. By using the proposed approach, we want to increase heart disease prediction accuracy and contribute to the creation of more effective clinical decision support systems.

Table 1 displays the parameters used to train and evaluate the self-attention-based Transformer model for heart disease prediction utilizing the Cleveland dataset. The model consists of an embedding layer, Transformer encoder layers, and a fully connected layer for classification. Iterate through the training dataset in mini-batches, compute the loss, backpropagate, and use the optimizer to update the model weights. After training the model, assess its performance on the testing set. Analyze the model’s predictions and interpret the learned patterns. Determine the self-attention mechanism’s key characteristics and attention weights. In this work, we carry out both binary and multi-class classification tasks in the experiments. For the binary classification problem, we predict the presence or absence of heart disease. For the four-class classification problem, we divide the labels into four unique classes to reflect various risk levels of heart disease. Table 2 displays the number of classes used in the experiments. In order to validate the self-attention-based Transformer model performance, we compared it with various baseline approaches.

**Table 1:** Parameters of the proposed model used in the experiments.

Parameter	Description
Model	Self-attention-based transformer model
Input dimension $=14$	Input features dimension
Output dimension $=\mathrm{2,4}$	Number of output classes
d-model = 128	Dimensionality of the model's hidden states
nhead $=4$	Attention heads in the multi-head self-attention
Num-layers $=4$	Layers in the encoder
Dropout $=0.2$	Dropout probability
Batch-size $=\mathrm{32,64}$	Number of samples
Epochs $=90$	number of iterations
Learning-rate $=0.001$	Learning rate
Optimizer $=$ Adam	optimizer used for updating the parameters
Train-loss	Avg raining loss over the training dataset
Cross entropy	Loss function
Test-loss	Avg loss over the testing dataset

**Table 2:** Binary and multiclass classification.

Class value	Description
Binary	Two class classification problem
Class 0	no heart disease
Class 1	presence of heart disease
Multi-class	four-class classification problem
Class 0	no heart disease
Class 1	low risk of heart disease
Class 2	moderate risk of heart disease
Class 3	high risk of heart disease

### Baseline approaches

We carried out a comparison study using a number of baseline methods frequently employed for heart disease prediction. We investigated CNN, RNN, RNN + (RNN with additional features), RETAIN (Reverse Time Attention Model), and Dipole as the baseline methods. We used an identical experimental design for each baseline strategy, including data preparation, model training, validation, hyperparameter adjustment, and assessment of the testing set.

#### CNN

Three convolutional layers make up the CNN model presented by Albelwi et al.^37^, which follows a standard neural network architecture. The kernel sizes for each convolutional layer range from 3 to 5, and each layer has 256 channels. In order to identify clinical data that was considerably class-imbalanced and forecast the development of coronary heart disease (CHD), a study done in^38^ developed an effective neural network using convolutional layers. For the purpose of predicting CVD, we classified the Cleveland dataset using this model. The size of the convolutional kernels is kept to (kernel-size = 3), and (pool-size = 2). The dropout rate for regularization is kept to (dropout-rate = 0.2).

#### RNN

One of the first models^39^ in the field of recurrent processing was introduced as LSTM, a sort of RNN with gated units. The input gate, forget gate and output gate make up the LSTM unit, a sequential architecture, commonly used in temporal data processing. These gates are essential for managing the information flow inside the LSTM unit. The LSTM unit uses a self-loop mechanism on its internal state as opposed to the recursive calculation method of conventional RNNs, which improves its capacity to retain and update information over time^40^. The input gate assesses the applicability of the current input and modifies the internal state in accordance with the system state at the previous time step. To begin with, we calculate the input embeddings, which are then passed into an LSTM layer. The hidden states generated by the LSTM are directly used by a linear classifier to predict the outcomes.

#### *RNN* + 

To improve performance or handle certain issues, RNN + refers to the expansion or combining of RNNs with other components. By integrating the hidden states, the RNN + extension of the RNN model incorporates a location-based attention mechanism into the output layer^41^. Encode the target categorization labels in the Cleveland dataset into a numerical representation so that the model can interpret it. We employ one-hot encoding for multi-class categorization.

#### RETAIN^42^

RETAIN is a state-of-the-art predictive model that leverages a two-level attention mechanism, enhancing both its functionality and interpretability. RNNs-like prediction accuracy is maintained by the unique neural attention model known as RETAIN, which is customized to enable thorough interpretation of prediction findings. The key characteristic of RETAIN is its attention mechanism, which emulates the clinical decision-making approach of doctors. The fundamental idea underlying RETAIN is to use context-level attention and time-level attention to describe the link between input sequences and the target variable. This attention mechanism allows RETAIN to draw attention to and weigh the important input sequence components, enabling a more in-depth comprehension of the model’s predictions. RETAIN exhibits performance that is comparable to RNNs and does not sacrifice prediction accuracy despite its interpretability.

#### Dipole^43^

Dipole employs a bi-directional RNN with three attention methods. In this case, we choose a variation of Dipole that has demonstrated superior performance. The embedding layer of the Dipole model is implemented as a multi-layer perceptron (MLP) with ReLu activation. They observed that, the local-based attention mechanism performs the best out of the three methods. Based on this discovery, we modify our model’s local-based attention mechanism to produce the final context vector that is used for prediction. The output of the bi-directional RNN with an attention layer is followed by a classification layer. This layer assigns the learned representations to the required classification labels and forecasts the probability for each class. This comparative analysis was conducted to assess the self-attention-based Transformer model’s performance against these standard methods. By comparing the Transformer model’s performance measures to those of the baselines, we were able to gain insight into the model’s strengths, shortcomings, and potential as an improved technique for heart disease prediction.

### Environment setting

The experiments of the proposed work are implemented using PyTorch 2.0. All training is carried out on a computer with an Intel Core i97900X processor, 128GB of RAM, 2 Nvidia Titan V graphics cards, and CUDA 9.0. For training our hypothetical model, we use Adam optimizer, with ${d}_{m}$, set to 128. We used the learning rate as, lr = 0.001 and the loss function as CrossEntropyLoss () to fine-tune the model. The time complexity of the proposed model is calculated as; $O\left({n}^{2}*d\right),$ where $n$ represents the sequence length, and $d$ reflects the dimensions of the hidden state.

### Evaluation metric

In this study, the classification tasks are measured using the accuracy metric. It calculates the percentage of properly identified examples in a dataset relative to all occurrences. In this particular case, the number of events for a particular user is expressed by the number of folds (k = 130). The remaining instances (k-1) serve as a training set for each iteration of the learning process, and the instance that is chosen serves as a test set. Then, the mean accuracy over all k trials is determined, such as;

6 $${\text{Accuracy}}=\frac{TTP+TTN}{TTP+TTN+FFP+FFN}$$

whereas FFP and FFN represent false positives and false negatives, respectively, TTP and TTN represent true positives and true negatives.

## Results and discussion

Compare the performance of the suggested Self-Attention-based Transformer Model to the baseline techniques. Determine which model has the best prediction accuracy and generalization capabilities by calculating the accuracy of each model. The experimental results achieved in the heart disease prediction task are shown in Table 3. The outcomes demonstrate how much better our suggested strategy is than all benchmark models, including RNN and RETAIN. Our solution surpassed these baseline models in terms of performance and predicted accuracy, which are commonly regarded as state-of-the-art approaches for heart disease prediction. Furthermore, we saw a wider performance disparity between our approach and the RNN-based model in our dataset. The table provides a comprehensive comparison of computing efficiency and accuracy among the different models considered as baselines. In order to validate the model performance on diverse dataset, we used the cardiovascular disease dataset, which is freely available on Kaggle. This dataset consists of 70,000 instances having 11 independent features. The computing time column specifically indicates the duration required to train each model once on the entire training dataset per epoch. As evident from the table, the proposed model exhibits faster training times in comparison to baseline models. The proposed model also achieves the highest accuracy of 95.2% using the cardiovascular disease dataset, shown in Table 4.

**Table 3:** Computation time and accuracy using Cleveland dataset.

Model	Computation time (s)	Accuracy $(\mathrm{\%})$
CNN	4.53	0.747
RNN	1.43	0.783
RNN +	3.52	0.871
RETAIN	4.45	0.850
Dipole	2.12	0.894
Proposed	1.90	0.965

**Table 4:** Computation time and accuracy using cardiovascular disease dataset.

Model	Computation time (s)	Accuracy $(\mathrm{\%})$
CNN	9.74	0.713
RNN	3.96	0.779
RNN +	6.38	0.863
RETAIN	7.12	0.832
Dipole	5.61	0.876
Proposed	3.57	0.952

**Figure 3:**
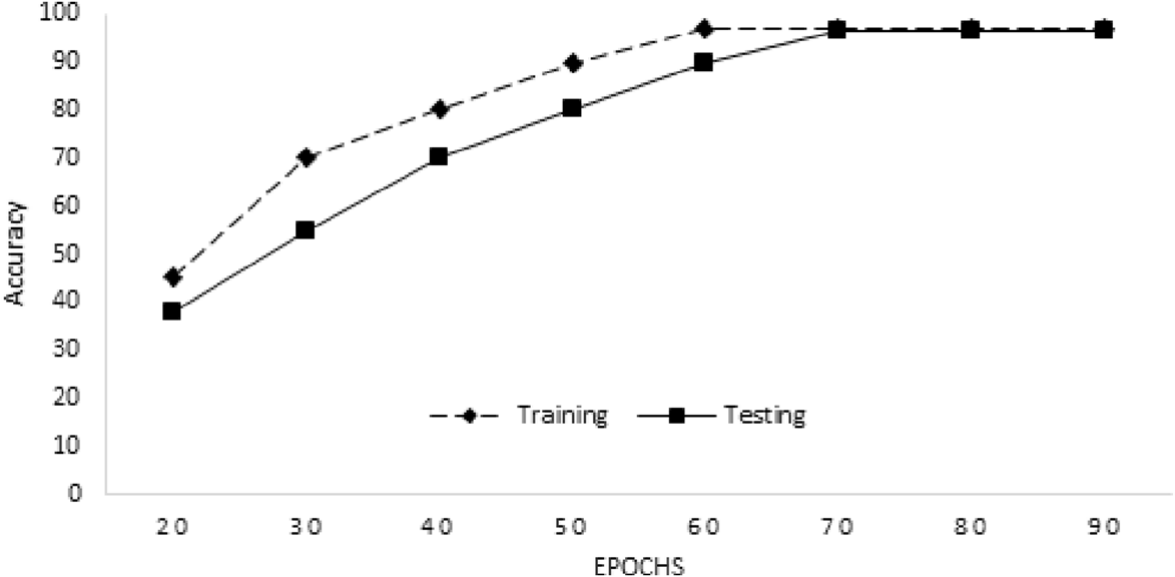
Training and testing accuracy of the model.

**Figure 4:**
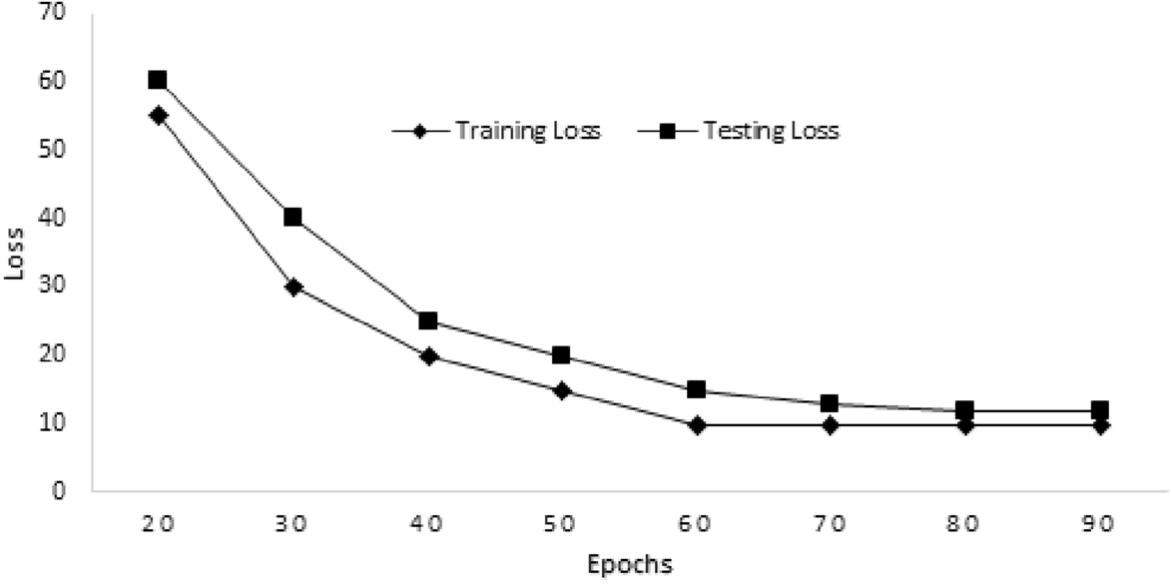
Training and testing loss of the model.

This advantage can be attributed to the straightforward and parallelizable structure of our suggested model. RNN models, on the other hand, encounter difficulties because of their sequential nature, leading to longer training durations, especially when working with datasets containing prolonged sequences. The suggested model’s interpretability when compared to RNN is also a key advantage. While RNN models are difficult to interpret, our proposed model provides more clarity and is simpler to understand. In healthcare applications, this interpretability can be quite helpful because it gives medical practitioners insights into the underlying causes of heart failure (HF) prediction and speeds up the decision-making process. Figure 3 represents the training and testing accuracy of the proposed model. We achieved 97.17% training accuracy and 96.51% testing accuracy by iterating the model for 90 epochs. Similarly, we get the minimum training loss of 0.10 and testing loss of 0.12, as shown in Fig. 4. In recent studies on CVD prediction using ML techniques, various classifiers have been employed^44^. Table 5 provides a summary of recent studies conducted for heart disease prediction, along with their achieved accuracy. The study conducted in^45^ deployed various classification techniques such as SVM, NB, and DT, for a CVD risk prediction. They achieved an accuracy of 90% for CVD risk prediction. Similar to this, the study conducted in^46^, described a prospective study with 423,604 subjects from the UK Biobank. For forecasting the risk of CVD, they unveiled an ML technique dubbed Auto-Prognosis. The work done in^47^ offered a novel approach for creating a predictive framework in the form of fuzzy methods to evaluate CVD risk using a neuro-fuzzy decision support mechanism. Their proposed approach intends to offer helpful assistance in determining the risk caused by cardiovascular diseases. Additionally, the research conducted in^48^ proposed the Gradient Boosting (GB) algorithm, which achieved an accuracy of 89.7%. Gradient Boosting uses a group of weak learners, which becomes computationally expensive when working with big datasets or complicated models. It is also sensitive to noise or outlier data. The maximum accuracy of 96.51% was achieved using the proposed model after data preprocessing, adjusting the input and output layers, incorporating more layers, and modifying the attention processes to collect relevant information.

**Table 5:** Comparison with various related studies.

Authors	Year	Approach	Accuracy (%)
Study^47^	2019	HRFLM	88.7
Study^46^	2021	NF model	91
Study^48^	2022	GBA	89.7
Study^45^	2023	NB SVM DT	90
Study^49^	2023	XGBH	80.6
Proposed	2023	Transformer model	96.51

The study conducted in^15^ deployed various ML algorithms for the task of heart disease prediction using the Cleveland database reflected in Table 6. They achieved a maximum accuracy of 90.78% using the K-NN algorithm. They realized that to enhance the precision of heart disease diagnosis, it is necessary to investigate cutting-edge methodologies and model fusions. Detecting CVD diseases such as heart attacks and coronary artery diseases are pivotal research problem. In a study conducted by^25^, the researchers utilized the Cleveland heart disease dataset to perform heart disease prediction. They deployed DT, RF, and a hybrid approach combining both algorithms. Through their heart disease prediction model, they achieved a higher accuracy of 88.7% using the hybrid approach. Heart disease may be quickly and inexpensively detected with the use of ML techniques. The research reported in^50^ suggests the use of an expert model called hyOPTXg to predict heart disease using an improved XGBoost classifier. On the Cleveland dataset, they achieved an accuracy of 94.7%. Heart disease prediction has gotten a lot of interest in the medical world. In the study^14^ a hybrid genetic algorithm (GA) and particle swarm optimization (PSO) optimized technique based on RF, named GAPSO-RF, is created and applied to identify the ideal features that can improve heart-disease prediction accuracy. On the Cleveland dataset, they obtained 95.6% accuracy in heart disease prediction. Their approach achieves good accuracy however, combining several techniques may increase the difficulty of parameter adjustment and convergence of optimization.

**Table 6:** Performance comparison on same dataset.

Authors	Year	Algorithm	Accuracy (%)
Study^15^	2020	NB, DT, RF, K-NN	90.78
Study^25^	2021	DT, RF, Hybrid	88.7
Study^50^	2022	hyOPTXg using XGBoost	94.7
Study^14^	2022	GAPSO-RF	95.6
Proposed	2023	Transformer model	96.51

The proposed strategy surpasses state-of-the-art approaches, with a remarkable accuracy of 96.51%. Th self-attention mechanism enables the model to effectively capture long-range relationships. The transformer model is able to address any point in the input sequence, unlike conventional sequential models like RNNs. It produces context-aware representations for each input token. Due to the attention mechanism, transformers provide efficient parallelization during training and inference. The model becomes more effective and scalable as a result of its parallelization capacity, especially when working with huge datasets. By allocating attention weights to various input places, the self-attention mechanism enables interpretability. This makes it possible to visualize the significance and relevance of particular characteristics in the prediction process.

The limitation of the proposed model is; It becomes difficult to understand the transformers architecture, particularly when it becomes deeper and complicated. Especially, to grasp how the model generates particular predictions or what sequence elements are essentials. To address this issue, we used attention visualization approaches, which gave us helpful insight about the framework's decision-making process.

## Conclusion

In this work, we developed a novel attention-based transformer model for the task of heart disease prediction. This model applied the strength of position-level attention mechanisms and self-attention layers to learn the representation of the complete sequence, in contrast to conventional RNN methods. Through the use of this distinct mechanism, we were able to identify and evaluate the relative weights of the various sequence components, improving the effectiveness of prediction. Beyond heart disease, a variety of clinical risk prediction tasks can be performed using the proposed technique due to its versatility. The fundamental advantage of this architecture is its well-designed network topology, which enables maximum parallelization. In contrast to RNN-based models, which suffer from sequential processing and limited parallelization, the proposed paradigm permits efficient and simultaneous computing across the whole sequence. The proposed model performs well in real-world circumstances using benchmark dataset and reduces training and inference times. To validate the performance, we conducted various experiments and compared their results with various related study to demonstrate that the proposed model is more accurate than cutting-edge methods. The proposed method is adaptable, which highlights its potential for usage in a range of healthcare contexts beyond heart disease prediction, providing informative data and assisting in decision-making.

In future, we want to Integrate transfer learning with the proposed model to enhance its performance, especially in the scenarios of dealing with limited labeled data.

## Data Availability

The datasets and code will be available from the corresponding author on request.
